# Does types of atrial fibrillation matter in the impairment of global and regional left ventricular mechanics and intra-ventricular dyssynchrony?

**DOI:** 10.3389/fcvm.2022.1019472

**Published:** 2022-10-24

**Authors:** Xiao-Wen Zhen, Wen-Cai Li, Hua Wang, Nian-Peng Song, Lin Zhong

**Affiliations:** ^1^Department of Diagnostics, Binzhou Medical University, Yantai, China; ^2^Department of Cardiology, Affiliated Yantai Yuhuangding Hospital, Qingdao University Medical College, Yantai, China

**Keywords:** atrial fibrillation, dyssynchrony, ventricular strain, heart rate, age

## Abstract

**Background:**

Atrial fibrillation (AF) is the most common sustained cardiac arrhythmia, which is associated with cardiac dysfunction. This study aimed to compare the impairment severity of left ventricular strain and intra-ventricular dyssynchrony using echocardiography-derived velocity vector imaging in patients with different types of AF without heart failure.

**Methods:**

168 non-valvular AF patients with normal left ventricular ejection fraction (98 paroxysmal AF patients and 70 persistent AF patients) and 86 healthy control subjects were included in this study. Regional and global left ventricular longitudinal and circumferential strain were measured. Time to regional peak longitudinal strain was measured and the standard deviation of all 12 segments (SDT-S) was used as a measure of intra-ventricular dyssynchrony.

**Results:**

Significantly lower GLS (−18.71 ± 3.00% in controls vs. −17.10 ± 3.01% in paroxysmal AF vs. −12.23 ± 3.25% in persistent AF, *P* < 0.05) and GCS (−28.75 ± 6.34% in controls vs. −24.43 ± 6.86% in paroxysmal AF vs. −18.46 ± 6.42% in persistent AF, *P* < 0.01) were observed in either persistent AF subjects or paroxysmal AF subjects compared with healthy control subjects (*P* < 0.05). The impairment was much worse in persistent AF subjects compared with paroxysmal AF subjects (*P* < 0.001). Intraventricular dyssynchrony was found in both persistent AF patients and paroxysmal AF patients, and it’s worse in persistent AF patients (52 ± 18 ms in controls, 61 ± 17 ms in paroxysmal AF, and 70 ± 28 ms in persistent AF, *P* < 0.05). Multivariate regression analysis revealed AF types were independent risk factors of GLS, GCS, and intraventricular dyssynchrony.

**Conclusion:**

AF types were not only associated with impaired longitudinal and circumferential left ventricle mechanics but also intra-ventricular mechanical dyssynchrony. Worse systolic mechanics and intra-ventricular dyssynchrony were found in patients with persistent AF compared with these in patients with paroxysmal AF.

## Introduction

Atrial fibrillation (AF) is the most common sustained cardiac arrhythmia. AF progresses from short, rare episodes, to longer and more frequent attacks (1). Irregular ventricular rates, decrease in coronary blood flow, long-term remodeling of the left ventricle (LV), and conditions associated with AF including aging, hypertension, coronary artery disease (CAD), and diabetes mellitus can harm the ventricular function (2). Reant et al. validated early longitudinal and circumferential LV systolic function abnormalities in patients with isolated paroxysmal AF but normal ejection fraction using 2-dimensional strain technique (3), but the difference of ventricular strain and dyssynchrony among patients with different types of AF was not clear, either in longitudinal and circumferential aspects or in global and regional aspects.

The factors influencing the systolic function of LV involve not only the global and regional contractile function but also the contractile pattern. Mechanical dyssynchrony is considered an independent predictor for adverse cardiovascular outcomes in patients with left ventricular dysfunction, heart failure, or both after myocardial infarction (4). Experimental and clinical reports have demonstrated that dyssynchrony results in decreased cardiac output and a reduced rate of left ventricular relaxation and filling (5, 6). Subclinical diastolic LV dysfunction is also found to be associated with mechanical LV dyssynchrony (7). The difference in ventricular function and dyssynchrony among patients with different types of AF was not clear.

Velocity vector imaging (VVI) possesses the angle-independent advantage and provides more accurate data on global and regional cardiac function and intra-ventricular dyssynchrony (8). Early detection of cardiac dysfunction and dyssynchrony with the longitudinal strain and the circumferential strain was shown in different clinical conditions (4, 8–14). Global longitudinal strain (GLS) provides independent and incremental prognostic information regarding the long-term risk of cardiovascular morbidity and mortality (15, 16).

**FIGURE 1:**
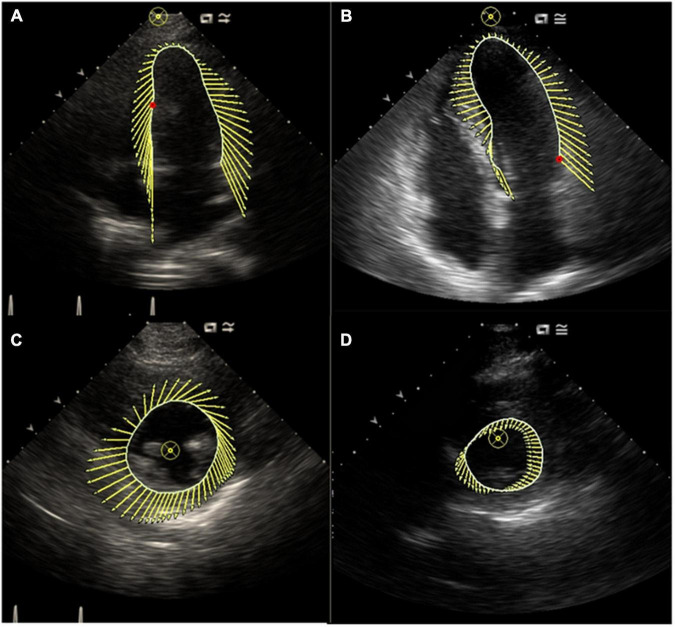
**(A)** Characterization of LV motion in the long axis in a control subject; **(B)** characterization of LV motion in the long axis in a patient with AF; **(C)** characterization of LV motion in the short axis in a control subject; **(D)** characterization of LV motion in the short axis in a patient with AF. This figure showed synchronous motion of LV segments in control subject and impaired synchronous motion of LV segments in a patient with AF.

**FIGURE 2:**
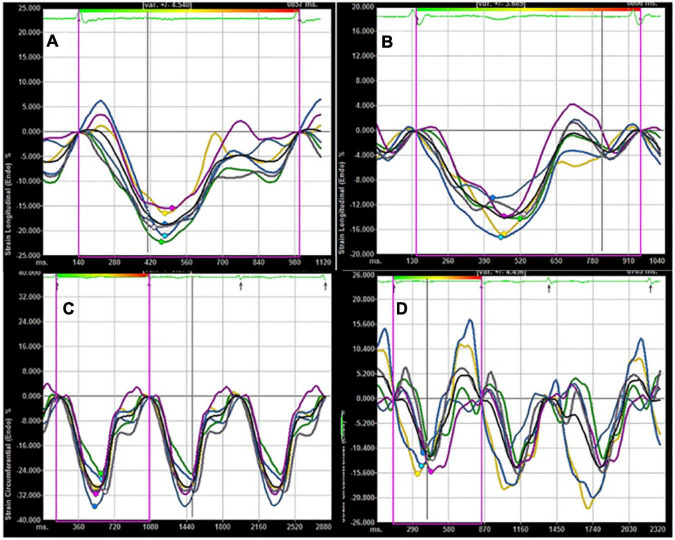
**(A)** Longitudinal LV strain in a control subject; **(B)** longitudinal LV strain in a patient with AF; **(C)** circumferential LV strain in a control subject; **(D)** circumferential LV strain in a patient with AF. Compared with control subject, lower absolute values of longitudinal and circumferential LV strain were found in a patient with AF.

In this cross-sectional study, we used VVI to compare the severity of both longitudinal and circumferential ventricular mechanics impairment and dyssynchrony in patients with different types of AF (Figures 1, 2). In addition, we analyzed each component of AF-associated conditions that influences ventricular dysfunction and dyssynchrony and determined the key factors among them.

## Materials and methods

### Study population

This study was approved by the ethics committee and the details of clinical and biological parameters were obtained. All participants provided their written informed consent to participate in this study. AF and CAD were defined according to published guidelines (1, 17, 18). Subjects with heart failure, moderate to severe valvular heart disease, primary myocardial, and pericardial diseases, myocardial infarction, left ventricular ejection fraction (LVEF) < 0.50, intraventricular block, bundle branch block, and echocardiography images that do not meet the analytical needs were excluded. Finally, 168 consecutive patients with non-valvular AF and 86 healthy control subjects were included in the study. The included 254 subjects were separated into 3 groups: the control group (Control, *n* = 86), persistent AF group (Pers AF, *n* = 70), and paroxysmal AF group (Paro AF, *n* = 98).

### Conventional echocardiography

All subjects underwent routine echocardiography examination in the left lateral decubitus position (Siemens ACUSON Sequoia 512). Left atrial and ventricular dimensions were measured according to the recommendations of the American Society for Echocardiography (19) and left ventricular mass was calculated using Devereux’s formula. Left ventricular mass and left atrial volume were indexed for body surface area (20). The LV volumes and LVEF were traced manually at end-diastole and end-systole at apical 4- and 2-chamber views and derived from modified biplane Simpson’s method. Pulsed-wave mitral inflow Doppler was obtained by placing the Doppler sample volume between the tips of the mitral leaflets. Tissue Doppler imaging mode was employed to measure the peak early diastolic velocity of the medial mitral valve ring at the basal septal segment (e’). The E/e’ ratio was obtained by dividing E by e’ (21).

### Velocity vector imaging

Standard grayscale 2 dimension images were acquired in the 2- and 4-chamber apical views as well as the parasternal short-axis views at the level of the papillary muscles. Digital cine loops were obtained at high frame rates (> 30 frames/s). The images were then exported to a personal computer and analyzed by an offline pixel-tracking software package (Velocity Vector Imaging, Siemens Medical Solutions, Mountain View, California). From an end-systolic single frame, a region of interest was traced on the endocardial cavity interface by a point-and-click approach. Further adjustment of the region of interest was performed to ensure that all of the myocardial regions were included. Strain curves were then computed automatically by tracking the motion of acoustic objects frame-by-frame. For the paroxysmal AF patients, all echocardiographic data were collected in sinus rhythm. For persistent AF patients, echocardiographic data were collected as a mean of at least 3 cardiac cycles in AF rhythm.

GLS for each patient were derived from the mean value of both apical 2- and 4-chamber views (a total of 12 segments automatically generated by the software), and the global circumferential strain (GCS) of each patient were composed of the mean value from 6 LV mid-wall short-axis segments. Time to regional peak longitudinal strain was measured and the SD of all 12 segments (SDT-S) was used as an index of intra-ventricular mechanical dyssynchrony. All echocardiographic analyses were performed by observers who were blinded at all times to the clinical data.

### Statistical analysis

**FIGURE 3:**
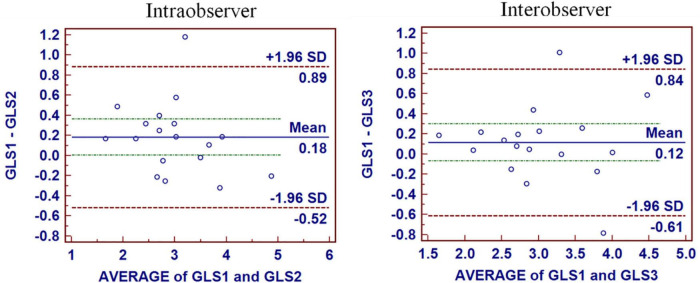
Intraobserver and interobserver reproducibility for GLS. The mean bias of intraobserver was 0.18 (limits of agreement, −0.52 to 0.89) for GLS. The mean bias of interobserver was 0.12 (limits of agreement, −0.61 to 0.84) for GLS.

The continuous variable data were tested for normality distribution using Kolmogorov-Smirnov and Shapiro-Wilk tests. Normal distribution parameters were presented as the mean ± standard deviation (*SD*) while the non-normal distribution parameters were expressed as median (25th and 75th interquartile). The independent-sample *t*-test was used for comparisons of normal distribution variables and the Mann-Whitney *U*-test was used for comparison of non-normal data. Categorical variables were compared using Fisher’s test or Pearson’s chi-square test and presented as absolute frequency and percentages. To compare the mean value of continuous variables between the groups with different types of AF, one-way ANOVA followed *post hoc* LSD *t*-test or Tamhane test was used. The student’s *t*-test was used in the subgroup comparison within different types of AF. The association of echocardiographic variables with clinical variables was assessed by Spearman correlation. The potential confounding factors that were relevant in the univariate analysis (*P* < 0.05) and AF type as a categoric value were included in the multiple variable stepwise regression analysis. Bland-Altman plots were used to assess the reproducibility for GLS of intra-observer and interobserver (Figure 3). For all statistical procedures, SPSS 18.0 statistical analysis software was used. A *p*-value < 0.05 was considered statistically significant.

## Results

### Patient characteristics

Age and gender were well balanced among groups. The Pers AF subjects and Paro AF subjects had a significantly higher systolic blood pressure (SBP) and prevalence of coronary artery disease (CAD), and heavier concentric obesity than controls (Table 1). Both the Pers AF and Paro AF patients had higher CHA_2_DS_2_-VASc Scores, higher prevalence of Hypertension and diabetes mellitus (DM), and higher percents of medication use including anticoagulant, beta-blockers, angiotensin-converting enzyme inhibitor/angiotensin II-receptor blocker (ACEI/ARB), calcium channel blocker (CCB) and statin.

**TABLE 1:** Comparison of clinical characteristics among subjects with different types of AF.

	Control	Pers AF	Paro AF
	
	*n* = 86	*n* = 70	*n* = 98
Gender (FM/M)	46/40	24/46	36/62
Age (years)	55.7 ± 8.1	59.5 ± 9.4	58.4 ± 10.8
SBP (mmHg)	124 ± 14	140 ± 20***	136 ± 19***
DBP (mmHg)	81 ± 12	84 ± 11	78 ± 13
HR (bpm)	64 ± 10	85 ± 19***	66 ± 11^###^
BMI (kg/m^2^)	24.39 ± 3.50	26.62 ± 3.40**	26.2 ± 3.69*
QRS width (ms)	86 ± 18	89 ± 19	88 ± 17
WC (cm)	86.07 ± 6.71	92.90 ± 7.80***	90.83 ± 10.09*
WHR	0.86 ± 0.05	0.91 ± 0.04***	0.91 ± 0.06***
TC (mmol/L)	4.86 ± 0.99	4.80 ± 1.51	4.72 ± 0.89
TG (mmol/L)	1.16 (0.90,1.45)	1.39 (0.99,1.88)	1.38 (1.02,2.12)
HDL-C (mmol/L)	1.34 ± 0.28	1.24 ± 0.30	1.28 ± 0.32
LDL-C (mmol/L)	2.61 ± 0.67	2.73 ± 1.11	2.62 ± 0.65
CAD (*N*, %)	0 (0)	30 (42.9)***	34 (34.7)**
DM (*N*, %)	0 (0)	30 (42.9)***	16 (16.3)**
Hypertension (*N*, %)	0 (0)	46 (62.7)***	54 (55.1)***
Obesity (*N*, %)	2 (4.7)	10 (14.3)*	12 (12.2)*
CHA_2_DS_2_-VASc score	1 (0,1)	2 (1,3)***	2 (1,3)***
Medication use			
Anticoagulant (*N*, %)	0 (0)	50 (71.4)***	56 (57.1)***
Beta-blocker (*N*, %)	0 (0)	52 (74.3)***	60 (61.2)***
ACEI/ARB (*N*, %)	0 (0)	44 (62.9)***	50 (51.0)***
CCB (*N*, %)	0 (0)	16 (22.9)***	20 (20.4)***
Statin (*N*, %)	0 (0)	30 (42.9)***	36 (36.7)***

SBP, systolic blood pressure; DBP, diastolic blood pressure; HR, heart rate; BMI, Body mass index; WC, waist circumference; WHR, waist-hip ratio; TC, total cholesterol; TG, total triglyceride; HDL, high-density lipoprotein cholesterol; LDL, low-density lipoprotein cholesterol; CAD, coronary artery disease; DM, Diabetes Mellitus. ACEI, angiotensin-converting enzyme inhibitor; ARB, angiotensin II-receptor blocker; CCB, calcium channel blocker Vs. Control, *P < 0.05, **P < 0.01, ***P < 0.001; vs. Pers AF, ^###^*P* < 0.001.

Compared with controls, Pers AF subjects had higher left ventricular mass index (LVMI), E/e’, and thicker ventricular septum. Paro AF subjects had significantly higher LV end-diastolic volume index (LVEDVI) and lower e’ than controls. LVMI of Paro AF subjects was higher than controls, but the difference was not significant (Table 2).

**TABLE 2:** Comparison of conventional echocardiographic parameters among subjects with different types of AF.

	Control	Pers AF	Paro AF
	
	*n* = 86	*n* = 70	*n* = 98
LVMI (g/m^2^)	117.17 ± 24.13	138.84 ± 42.54*	128.35 ± 35.03
LVIDd (mm)	43.91 ± 3.74	46.41 ± 6.44*	44.94 ± 4.65
IVST (mm)	10.91 ± 1.43	12.26 ± 1.70**	11.80 ± 2.09
PWT (mm)	10.14 ± 1.48	11.02 ± 1.72	10.80 ± 1.77
LAVI (ml/m^2^)	27.23 ± 12.65	34.43 ± 14.75*	30.82 ± 11.73
LVEF	0.61 ± 0.05	0.59 ± 0.04	0.61 ± 0.07
LVEDVI (ml/m^2^)	26.44 ± 6.08	26.82 ± 9.03	29.79 ± 7.89*
LVESVI (ml/m^2^)	8.95 (8.21,11.15)	9.97 (8.28,12.74)	10.34 (8.50,13.35)
E (m/s)	0.71 ± 0.13	0.88 ± 0.22**	0.68 ± 0.18^###^
e’(m/s)	0.11 ± 0.02	0.12 ± 0.03	0.10 ± 0.02**^##^
E/e’	6.33 ± 1.35	8.09 ± 3.15*	7.24 ± 2.41

LVMI, left ventricular mass index; LVIDd, left ventricular diameter in end diastole; IVST, interventricular septum thickness; PWT, left ventricular posterior wall thickness; LAVI: left atrial volume index; LVEF, left ventricular ejection fraction; LVEDVI, LV end-diastolic volume index; LVESVI, LV end-systolic volume index; E, maximum amplitudes of the early diastolic wave; e’, peak early diastolic velocity of septal mitral valve ring. Vs. control, *P < 0.05, **P < 0.01; vs. Pers AF, ^##^*P* < 0.01, ^###^*P* < 0.001.

### Comparison of regional longitudinal and circumferential strain

Pers AF subjects had worse regional longitudinal mechanics compared with both controls and Paro AF subjects. All the segmental longitudinal strains except for the apical lateral segment of Pers AF and 4 segmental longitudinal strains of Paro AF were significantly lower than controls, although LVEF did not differ significantly between Paro AF subjects, Pers AF subjects, and controls. Except for the apical inferior segment and apical anterior segment, all the other segmental longitudinal strain was lower in Pers AF than in Paro AF subjects (Table 3).

**TABLE 3:** Comparison of regional longitudinal strain (%) among subjects with different types of AF.

	Control	Pers AF	Paro AF
	
	*n* = 86	*n* = 70	*n* = 98
Basal septum	−15.88 (11.28,18.59)	−8.89 (5.33,12.53)*	−13.50 (6.84,17.12)*^#^
Middle septum	−19.25 ± 4.61	−9.02 ± 4.12***	−15.80 ± 5.41***^###^
Apical septum	−19.81 ± 6.86	−11.57 ± 5.27***	−17.47 ± 7.06^###^
Basal lateral	−23.21 ± 6.30	−16.45 ± 6.42***	−19.77 ± 8.02*^#^
Middle lateral	−16.76 ± 5.09	−11.83 ± 4.99***	−15.23 ± 6.84^#^
Apical lateral	−14.72 ± 6.07	−11.43 ± 6.10	−15.34 ± 7.66^#^
Basal inferior	−17.92 ± 6.67	−11.72 ± 6.30***	−16.62 ± 6.75^##^
Middle inferior	−19.26 ± 5.30	−10.44 ± 3.87***	−16.15 ± 5.00*^###^
Apical inferior	−20.91 (13.43,28.76)	−12.33 (9.65,20.94)*	−18.22 (13.16,22.37)
Basal anterior	−21.13 ± 7.78	−15.37 ± 7.16**	−20.80 ± 8.06^##^
Middle anterior	−16.34 ± 5.94	−11.90 ± 5.31**	−16.92 ± 5.58^###^
Apical anterior	−18.11 ± 9.69	−12.03 ± 8.11**	−15.81 ± 6.03

Vs. control, *P < 0.05, **P < 0.01, ***P < 0.001; vs. Pers AF, ^#^*P* < 0.05, ^##^*P* < 0.01, ^###^*P* < 0.001.

Pers AF subjects also had lower regional circumferential strain compared with either controls or Paro AF subjects. All the segmental circumferential strains of Pers AF and 4 segmental circumferential strains of Paro AF were significantly lower than controls. Except for the middle lateral segment, middle inferior segment, and middle posterior segment, all the other 3 segmental circumferential strains were lower in Pers AF than in Paro AF subjects (Table 4).

**TABLE 4:** Comparison of regional circumferential strain (%) among subjects with different types of AF.

	Control	Pers AF	Paro AF
	
	*n* = 86	*n* = 70	*n* = 98
Middle anteroseptum	−36.03 ± 8.42	−21.02 ± 9.60***	−31.07 ± 11.97*^###^
Middle anterior	−28.08 ± 10.16	−19.43 ± 9.20**	−26.23 ± 11.71^#^
Middle lateral	−27.24 (18.10,35.60)	−21.16 (10.39,26.77)*	−21.28 (12.77,28.49)*
Middle posterior	−25.48 ± 8.89	−17.67 ± 9.76***	−20.03 ± 9.35*
Middle inferior	−25.27 ± 9.40	−16.62 ± 7.34***	−20.53 ± 7.23*
Middle septum	−34.11 (24.90,38.21)	−17.08 (9.02,24.16)*	−27.85 (20.55,33.79)^#^

Vs. control, *P < 0.05, **P < 0.01, ***P < 0.001; vs. Pers AF, ^#^*P* < 0.05, ^###^*P* < 0.001.

### Comparison of global longitudinal strain, global circumferential strain, and intra-ventricular dyssynchrony

**FIGURE 4:**
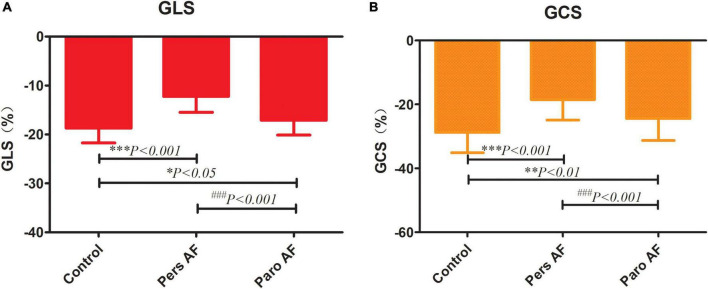
Comparison of GLS **(A)** and GCS **(B)** among control subjects and subjects with different types of AF. GLS, global longitudinal strain; GCS, global circumferential strain; GLS (−18.71 ± 3.00% in controls, −17.10 ± 3.01% in Paro AF, −12.23 ± 3.25% in Pers AF) GCS (−28.75 ± 6.34% in controls, −24.43 ± 6.86% in paroxysmal AF, −18.46 ± 6.42% in persistent AF) vs. control, *P < 0.05, **P < 0.01, ***P < 0.001; vs. pers AF, ^###^*P* < 0.001.

**FIGURE 5:**
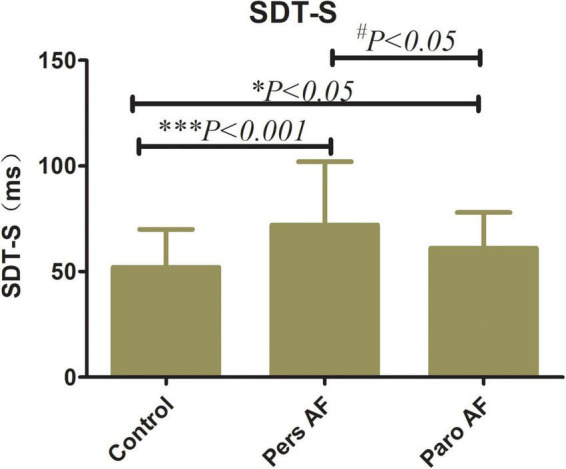
Comparison of SDT-S among control subjects and subjects with different types of AF SDT-S, *SD* of time to peak longitudinal strain of all 12 segments SDT-S (52 ± 18 ms in controls, 61 ± 17 ms in Paro AF, and 70 ± 28 ms in Pers AF) vs. Control, *P < 0.05, ***P < 0.001; vs. Pers AF, ^#^*P* < 0.05.

Both the Paro AF and Pers AF subjects had significantly lower GLS (−18.71 ± 3.00% in controls vs. −12.23 ± 3.25% in Pers AF, *P* < 0.001; −18.71 ± 3.00% in controls vs. −17.10 ± 3.01% in Paro AF, *P* < 0.05) than controls. GCS (−28.75 ± 6.34% in controls vs. −18.46 ± 6.42% in Pers AF, *P* < 0.001; −28.75 ± 6.34% in controls vs. −24.43 ± 6.86% in Paro AF, *P* < 0.01) was also significantly lower in Paro AF and Pers AF subjects compared with controls. Furthermore, the GLS and GCS were lower in Pers AF subjects than in Paro AF subjects (*P* < 0.001; *P* < 0.001) as shown in Figure 4. LV dyssynchrony was found to be worse in both Paro AF and Pers AF subjects (52 ± 18 ms in controls, 61 ± 17 ms in Paro AF, and 70 ± 28 ms in Pers AF, *P* < 0.05). SDT-S, as a measure index of LV dyssynchrony, was significantly higher in Pers AF than in Paro AF (*P* < 0.05) (Figure 5).

### Determinants of global longitudinal strain, global circumferential strain, and intra-ventricular dyssynchrony

To determine whether CAD is the key factor to cause impaired LV mechanics and synchronicity, in other words, whether impaired LV mechanics and synchronicity is mainly caused by CAD, we divided Pers AF patients and Paro AF patients into two subgroups, without CAD (*n* = 40, *n* = 64) and CAD (*n* = 30, *n* = 34), respectively. GLS, GCS, and SDT-S were compared between subgroups within Pers AF patients and Paro AF patients, but no significant difference was observed (Table 5).

**TABLE 5:** Subgroup comparison of VVI-derived parameters between subjects with and without CAD within different types of AF.

	Pers AF			Paro AF		
		
	Without CAD (*n* = 40)	CAD (*n* = 30)	*P*	Without CAD (*n* = 64)	CAD (*n* = 34)	*P*
GLS (%)	−12.14 ± 3.29	−12.39 ± 3.35	0.85	−17.39 ± 3.13	−16.38 ± 2.69	0.34
GCS (%)	−17.51 ± 5.79	−19.88 ± 7.30	0.33	−24.09 ± 7.07	−25.13 ± 6.64	0.67
SDT-S (ms)	70 ± 21	76 ± 39	0.59	59 ± 18	67 ± 15	0.22

GLS, global longitudinal strain; GCS, global circumferential strain; SDT-S, SD of time to peak longitudinal strain of all 12 segments; CAD, coronary artery disease.

Univariate analysis with Spearman correlation showed heart rate (HR) (*r* = 0.406, *P* < 0.001), SBP (*r* = 0.257, *P* = 0.009), body mass index (BMI) (*r* = 0.242, *P* = 0.014), and LVMI (*r* = 0.218, *P* = 0.028) were risk factors of GLS while HR (*r* = 0.419, *P* < 0.001) was risk factor of GCS (Table 6). Stepwise multivariate regression analysis revealed only AF type and HR were independent risk factors of GLS and GCS (Table 7). That meant the absolute values of strain decrease with the increase of the different risk factors. Univariate correlation analysis showed age (*r* = 0.260, *P* = 0.007) correlated with SDT-S (Table 6). Stepwise multivariate regression analysis revealed AF types and age were independent risk factors of LV dyssynchrony (Table 7).

**TABLE 6:** Univariate analysis of GLS, GCS and SDT-S with Spearman correlation.

	GLS		GCS		SDT-S	
			
	r	*P*	r	*P*	r	*P*
HR (bpm)	0.406	<0.001	0.419	<0.001	0.091	0.351
Age (years)	0.024	0.803	0.060	0.548	0.260	0.007
SBP (mmHg)	0.257	0.009	0.198	0.051	0.147	0.147
DBP (mmHg)	0.188	0.060	0.003	0.975	0.034	0.736
BMI (kg/m^2^)	0.242	0.014	0.076	0.460	0.063	0.534
WHR	0.187	0.062	0.191	0.062	0.111	0.275
LVMI (g/m^2^)	0.218	0.028	0.179	0.079	0.116	0.252

GLS, global longitudinal strain; GCS, global circumferential strain; SDT-S, SD of time to peak longitudinal strain of all 12 segments; HR, heart rate; SBP, systolic blood pressure; DBP, diastolic blood pressure; BMI, Body mass index; WHR, waist-hip ratio; LVMI, left ventricular mass index.

**TABLE 7:** Stepwise multivariate regression analysis of GLS, GCS and SDT-S.

	B	β	Adjusted *R*^2^	*P*
**GLS (%)**				
AF type (0, 1, 2)	3.682	0.388	0.336	<0.001
HR (bpm)	0.136	0.304	—	0.004
**GCS (%)**				
AF type (0, 1, 2)	2.74	0.537	0.385	<0.001
HR (bpm)	0.044	0.167	—	0.049
**SDT-S (ms)**				
AF type (0, 1, 2)	9.75	0.333	0.161	<0.001
Age (years)	0.525	0.209	—	0.013

GLS, global longitudinal strain; HR, heart rate; GCS, global circumferential strain; SDT-S, SD of time to peak longitudinal strain of all 12 segments; AF type: 0 for no atrial fibrillation, 1 for paroxysmal atrial fibrillation and 2 for persistent atrial fibrillation.

Intraobserver and interobserver reproducibility for GLS were presented in Figure 3. The mean bias of intraobserver was 0.18 (limits of agreement, −0.52 to 0.89) for GLS. The mean bias of interobserver was 0.12 (limits of agreement, −0.61 to 0.84) for GLS. The intraclass correlation coefficients of interobserver variability and intra-observer variability were 0.960 and 0.888, respectively.

## Discussion

The results of the current study showed that AF types were not only associated with impaired LV longitudinal and circumferential strain but also intra-ventricular mechanical dyssynchrony. Early-stage LV mechanics impairment was found in patients with AF even when LVEF was still normal. Worse LV strain was found in patients with persistent AF than in patients with paroxysmal AF. That may mean when the AF progresses from short, rare episodes, to longer and more frequent attacks, LV mechanics and mechanical dyssynchrony take a turn for the worse.

Factors affecting hemodynamic function in patients with AF involve loss of coordinated atrial contraction, high ventricular rates, irregularity of the ventricular response, and decrease in myocardial blood flow, as well as long-term alterations such as atrial and ventricular cardiomyopathy (2, 22). It has been validated that heart failure is the strongest risk factor for the development of AF and AF may precipitate or exacerbate LV dysfunction (23, 24). In a research investigating left ventricular dysfunction in AF with GLS in a large population and a broad clinical spectrum (25), results showed that compared with an independent, non-AF cohort, with fully matched clinical features, and the same LVEF level, the AF cohort showed substantial GLS reduction. This result is consistent with our results.

Patients with AF are at a higher risk of developing heart failure. Several cardiovascular risks, including aging, hypertension, diabetes mellitus, obesity and metabolic abnormalities combined with a pro-inflammatory status are all notable clinical risk factors for AF development and LV dysfunction. These risk factors may contribute to the pre-clinical ventricular dysfunction in AF. Reduced GLS reflecting sub-clinical myocardial injury may occur prior to marked ventricular chamber dilation and might able to reflect subclinical systolic dysfunction and myocardial stiffness in paroxysmal AF (26). Previous studies have proposed that AF, even in patients with a preserved LVEF, is characterized by impaired intrinsic systolic properties measured with GLS, compared with those in sinus rhythm (27, 28).

The segmental ventricular strain also plays an important role in the early diagnosis and prognosis of multiple heart diseases (29–33). To find out whether the effect of AF on strain was related to segments, we performed segment analysis of regional longitudinal and circumferential strain. The results revealed impairment of regional longitudinal strain occurred in almost every segment of LV along with the AF development from paroxysmal to persistent. The significant impairment of regional circumferential strain occurred in 3 segments of the all 6 segments: Anteroseptal, anterior, and septal parts of LV along with the AF development from paroxysmal to persistent, but we can see an obvious aggravated trend from paroxysmal to persistent AF in the mean values of each part (for example Middle inferior part: −16.62 ± 7.34 in Pers AF and −20.53 ± 7.23 in Paro AF).

Although intra-ventricular dyssynchrony has been reported in heart failure, hypertrophic cardiomyopathy, and myocardial infarction patients (4, 34–36), and irregular ventricular rate may do harm to left ventricular synchronicity, intra-ventricular dyssynchrony in AF patients has not been reported. In this study, we found intra-ventricular mechanical dyssynchrony in patients with different AF types and worse intra-ventricular dyssynchrony in patients with persistent AF compared with these in patients with paroxysmal AF. Atrial fibrillation types were independent risk factors of intra-ventricular dyssynchrony and age was another independent risk factor of intra-ventricular dyssynchrony.

To date, there is still no uniform definition of intra-ventricular dyssynchrony and the mechanism is not yet clear (37–39). One cardiovascular magnetic resonance study revealed LV myocardium regional variation in interstitial fibrosis is a major determinant of LV intra-ventricular dyssynchrony irrespective of the LV global function (40). That may partly explain the mechanism by which intra-ventricular dyssynchrony occurs in AF patients because multiple AF related risk factors may causes interstitial fibrosis (41). Aging was tightly associated with cardiac fibrosis, which may cause loss of side-to-side fiber coupling and increases the transverse resistance and alters the properties of transverse propagation (42, 43). In this study, we also found that age was an independent risk factor of intra-ventricular dyssynchrony.

The myofiber geometry of the left ventricle changes gradually from a right-handed helix in the subendocardium to a left-handed helix in the subepicardium (44). Therefore, accurate assessment of cardiac mechanics has proven elusive to traditional imaging modalities, partly due to the complex spatial orientation and distribution of muscle fibers in the longitudinal and circumferential direction (45, 46). Quantification of LVEF based on ventricular volumes has been the primary method for assessing myocardial systolic function. But this measure is load-dependent and cannot early detect cardiac function impairment (47). VVI derived strain and strain rate accurately reflect intrinsic measures of myocardial contractility and enable early quantification of regional myocardial deformation for analysis of longitudinal and circumferential cardiac mechanics (48–50).

AF is associated with a variety of cardiovascular conditions such as aging, hypertension, heart failure, coronary artery disease, and diabetes mellitus, which have an additive effect on the perpetuation of AF by promoting a substrate that maintains AF. Conditions associated with AF not only simply serve as the causative factors, but also play the role as the markers for global cardiovascular risk as well as cardiac damage (22). LVMI, which is a risk factors of reduced ventricular strain, has been confirmed in multiple studies about hypertension (51, 52). In this study, we also found that LVMI was related to GLS and GCS, which is consistent with previous studies, but LVMI is not one of the major factors influencing strain reduction in the multiple regression analysis. In this study, we found that HR was an independent risk factor of both longitudinal and circumferential LV mechanics, and age was an independent risk factor of intra-ventricular dyssynchrony.

Recently, Reant et al. validated early longitudinal and circumferential LV systolic function abnormalities in patients with isolated paroxysmal AF but normal ejection fraction using a 2-dimensional strain technique (3). In our study, impaired longitudinal and circumferential LV systolic mechanics were found in both persistent and paroxysmal AF patients with normal EF. Although LVEF and E/e’ did not differ significantly, significant intra-ventricular dyssynchrony and reduction of GCS and GLS were found in paroxysmal AF patients. That might mean that these VVI derived variables are capable of early detection of ventricular dysfunction and dyssynchrony in AF patients.

### Study limitations

This is a relatively small study and further confirmation is needed in larger investigations. The multivariate analysis are possibly biased considering the lack of power and the study design. It therefore represents rather a statistical trend. VVI based on pixel-tracking technique relies on above-average image quality for good data extraction. The speckle pattern throughout the cardiac cycle, out-of-plane motion, reverberations, dropouts, and the differences between regional resolutions are all factors that might alter the real values of VVI derived variables.

## Conclusion

AF types were not only associated with impaired longitudinal and circumferential left ventricle mechanics but also intra-ventricular mechanical dyssynchrony. Worse systolic mechanics and intra-ventricular dyssynchrony were found in patients with persistent AF compared with these in patients with paroxysmal AF.

## Data availability statement

The raw data supporting the conclusions of this article will be made available by the authors, without undue reservation.

## Ethics statement

The studies involving human participants were reviewed and approved by the Ethics Committee of Yantai Yuhuangding Hospital. The patients/participants provided their written informed consent to participate in this study.

## Author contributions

X-WZ, N-PS, and LZ conceived and designed the experiments. N-PS, X-WZ, W-CL, LZ, and HW performed the experiments and analyzed the data. N-PS, LZ, and X-WZ wrote the manuscript. N-PS, LZ, X-WZ, W-CL, and HW revised the manuscript. All authors contributed to the article and approved the submitted version.

## Abbreviations

AF: atrial fibrillation
SD: standard deviation
SDT-S: standard deviation of time to regional peak longitudinal strain of all 12 segments
GLS: global longitudinal left ventricular strain
GCS: global left ventricular circumferential strain
LV: left ventricle
VVI: velocity vector imaging
LVEF: left ventricular ejection fraction
SBP: systolic blood pressure
DBP: diastolic blood pressure
WHR: waist-hip ratio
CAD: coronary artery disease
LVEDVI: left ventricle end diastolic volume index
LVESVI: left ventricle end systolic volume index
LVMI: left ventricular mass index
BMI: body mass index
HR: heart rate
Pers AF: persistent atrial fibrillation
Paro AF: paroxysmal atrial fibrillation
WC: waist circumference
TC: total cholesterol
TG: total triglyceride
HDL: high-density lipoprotein cholesterol
LDL: low-density lipoprotein cholesterol
DM: Diabetes Mellitus
LVIDd: left ventricular diameter in end diastole
LAVI: left atrial volume index
IVST: interventricular septum thickness
PWT: left ventricular posterior wall thickness
E: maximum amplitudes of the early diastolic wave
e’: peak early diastolic velocity of septal mitral valve ring.
